# Instrumental and Hedonic Motives for Emotion Regulation in Musical Practice

**DOI:** 10.3389/fpsyg.2021.643974

**Published:** 2021-06-30

**Authors:** Gerard Breaden Madden, Hans-Christian Jabusch

**Affiliations:** Institute of Musicians’ Medicine, University of Music Carl Maria von Weber, Dresden, Germany

**Keywords:** musical practice, emotion, emotion regulation, meta-emotion beliefs, goal orientation

## Abstract

Emotion regulation literature often emphasizes that individuals regulate their emotions for hedonic reasons. However, there is increasing support for an instrumental approach to emotion regulation. This approach suggests that emotions are regulated if they are believed to be beneficial to the pursuit of personally relevant goals. When pursuing a long-term goal, an individual may forego immediate, hedonic emotional reward in order to maximize the instrumental benefits of emotions. The current study investigates emotion regulation behaviour in the context of musical practice. We examine whether musicians adopt specific, regulated emotional stances which support their goal orientation, and which are in line with their beliefs regarding the functional impact of emotions. Via an online questionnaire, 421 musicians reported their goal-orientation, meta-emotion beliefs, and affect-regulation strategies. Participants then completed a scale assessing specific emotions they would regulate in order to support their musical practice. Data were analysed using PCA, MANOVA, subgroup analysis and categorical regression. Musicians reported using affect-improvement strategies more often than affect-worsening strategies in order to influence how they felt during musical practice. Greater reported use of affect-worsening strategies was associated with stronger meta-emotion beliefs supporting the possible instrumental benefits of unpleasant emotions (*F* = 30.33; *p* < 0.01; $\eta_{\text{p}}^{2}$ = 0.06). Musicians who strongly endorsed this belief more strongly pursued mastery goals in contrast to enjoyment goals. In terms of specific targeted emotions, musicians generally sought to reduce unpleasant emotions, and increase pleasant, energizing emotions in order to support their musical practice. However, a subgroup of mastery- rather than enjoyment-oriented musicians may seek a mixed emotional state, increasing anger and nervousness in conjunction with a number of pleasant emotions (Wilks λ_1,420_ = 14.42; *p* < 0.01; $\eta_{\text{p}}^{2}$ = 0.50). Musicians who pursue expert musical skills may be motivated to experience emotions that combine the instrumental and hedonic benefits of emotions. Musicians who practice for enjoyment may prioritize emotions that maximize only the hedonic benefits. Future research should aim to identify the regulated emotional states that best support specific musical practice outcomes in an individual. It will also be important to understand on all levels, including music performance quality as well as health and well-being, the outcomes that may be associated with the use of affect-worsening strategies and unpleasant emotions. Research in this field may equip musicians with novel skills for better pursuit of their goals, and may help to maximize health and well-being in musical practice.

## Introduction

The management of our emotions is an integral part of our lives. Individuals monitor their emotions and develop regulation strategies in order to adjust their emotions to a desirable or preferred state (Lane et al., 2011). As it is well-established that emotions can play a positive and adaptive role in our thoughts and behaviours, the process of emotion regulation allows us to function more effectively (Gross, 1999; Hanin, 2010). For example, emotions can improve our motivation, engagement and proactivity, prepare us for rapid motor responses, and adapt our learning styles according to situational demands (e.g., Fridja, 1986; Clore, 1994; Cahill and van Stegeren, 2003; Foo et al., 2009; Bledow et al., 2011; Mazur and Laguna, 2019). Evidence also suggests that emotions are predictive of our performance (Beedie et al., 2000). If emotions predict our performance and impact our ability to function effectively, then regulating emotions should be seen as an important psychological skill (Lane, 2012). Furthermore, individuals may benefit from understanding which emotions should be regulated in a given circumstance, and whether these emotions should be increased or decreased (Lane, 2012). Literature on emotion regulation has often emphasized the importance of reducing unpleasant emotional experiences (Gross, 1999). This emphasis is understandable, as unpleasant emotions are predictors of mental health (Tamir et al., 2008). However, the field of emotion regulation has expanded over the last number of decades to address the many ways in which emotions are experienced and expressed. With this expansion, it is increasingly clear that emotion regulation involves the increasing and decreasing of both positive and negative emotions (Parrott, 1993). One enduring interest of emotion researchers is to better understand how emotions are regulated in different applied contexts. One context that seems particularly relevant to study is musical practice. Making music on a professional level is considered to be one of the most complex of human accomplishments (Münte et al., 2002). Musical practice involves the processing, integration, and coordination of multisensory information, and it requires the development of highly complex motor programs with maximum spatio-temporal demands. Furthermore, the behaviours and strategies used in musical practice vary in sophistication and diversity as a musician progresses in skill (Gruson, 1988; Hallam, 2001; Lehmann and Papoušek, 2003). These characteristics make musical practice an ideal context to study from many perspectives including motor learning, expertise development, musical pedagogy, among others. However, musical practice has received limited research attention with respect to emotion regulation. The following section will outline research and findings that are relevant to studying emotion regulation in a musical practice setting.

### Hedonism and Utility in Emotion Regulation

Research suggests that emotion regulation is primarily driven by immediate affective considerations (Larsen, 2000; Tamir, 2005). Specifically, individuals are motivated to regulate their emotions in order to maximize feeling good, and minimize feeling bad. This process is generally referred to as *hedonic emotion regulation* (e.g., Tamir et al., 2008). However, it has been argued that this hedonic principle does not sufficiently explain all regulatory efforts (Erber and Erber, 2000). Although individuals may sometimes wish to maximise emotional pleasure, they may also choose certain emotional states for reasons other than immediate hedonic outcomes (Tamir, 2005). In addition to their hedonic quality, emotions also possess functional characteristics. Therefore, while some emotions may be pleasant to experience, one reason for individuals to regulate their emotions is that emotions can be useful (Fridja, 1986; Tamir, 2009). It follows then that individuals can be motivated to achieve positive outcomes rather than experiencing positive emotions. The process by which individuals experience useful emotions in order to facilitate positive outcomes is generally referred to as *instrumental emotion regulation* (e.g., Tamir et al., 2008; Lane et al., 2011).

There is increasing empirical support for the instrumental emotion regulation process. Findings on this topic broadly suggest that individuals may be motivated to experience whatever type of affect is believed to lead to instrumental success (Tamir et al., 2007; Tamir, 2009; Lane et al., 2011). Sport contexts provide perhaps the most obvious examples of instrumental regulation. As success is commonly considered an important outcome in sport, athletes readily seek out whatever emotions they believe will benefit their performance and ultimately result in success (Lane, 2008; Lane et al., 2011; Cooper et al., 2018). Generally, athletes prefer positive, motivating, and high-energy emotions. This type of affect may indeed facilitate better performance (Lane et al., 2012). Positive affect has been shown to support better decision making (Carnevale and Isen, 1986) and is helpful for tasks involving creativity and innovation (Isen et al., 1987; Estrada et al., 1994). In addition to affect, positive attitudes toward a task may also improve how readily and well that task is undertaken. For example, Spector et al. (2014) showed that positive attitudes toward musical practice were associated with better motor performance in child pianists’ scale playing.

Although positive affect may be beneficial to performance and practice, the association between emotions and performance is highly individualized and does not depend on whether an emotion is positive or negative (Hanin, 2010). Indeed, some individuals may believe that positive affect does not always facilitate better performance (Lane, 2012). For example, in a video game scenario, participants were motivated to experience anger when they anticipated a confrontational task for which anger was believed to be useful. In this scenario, anger was shown to improve task performance (Tamir et al., 2008). Additionally, Lane et al. (2011) reported that 15% of runners believed that unpleasant emotions such as anger and anxiety would help to improve their running performance. Typically, unpleasant emotions are not associated with better performance and practice. Despite this, Lane (2012) notes a mixed body of evidence on this topic. Certain kinds of negative affect are thought to intensify an individual’s effort to pursue their preferred outcome (Mazur and Laguna, 2019). Moreover, some athletes believe that extreme levels of unpleasant emotions are actually necessary in order to cope with the physical demands of a task (Hanton et al., 2008).

Beliefs regarding the functional impact of emotions in different contexts are generally referred to as *meta-emotion beliefs* (Hanin, 2010; Lane et al., 2011). These beliefs are knowledge regarding the utility of different emotions that individuals develop from previous experiences in their lives. If individuals previously experienced a particular emotion in a situation in which they performed successfully, then this emotion may be thought of as having facilitated their success. Understandably, individuals may wish to replicate this success in the future. Once an emotion has been associated with successful goal attainment, there is a greater chance that this emotion will re-emerge in similar situations (Hanin, 2010). Meta-emotion beliefs are therefore thought to influence the choice of emotions that individuals seek to regulate (Lane et al., 2011). Athletes who reported meta-emotion beliefs supporting the utility of unpleasant emotions also reported using strategies to increase the intensity of these emotions (Lane et al., 2011; Robazza et al., 2004). Expecting unpleasant emotional experiences to produce positive outcomes may seem counterintuitive. However, Lane et al. (2011) note that if an unpleasant emotion becomes associated with a positive outcome (such as successfully attaining sporting success), then an individual may feel that experiencing an unpleasant emotion can result in a pleasant experience. Therefore, depending on the activity and the expected benefits, unpleasant emotions may promote certain positive outcomes in ways that positive affect cannot (Davis et al., 2010). This raises the question of what outcomes can be promoted by different emotions?

### Short-Term vs. Long-Term Goals

In everyday life, the emotions that are pleasant to experience may sometimes overlap with the emotions that are useful to experience. It follows then that individuals may regulate their emotions in order to maximise either hedonic rewards or instrumental rewards, but also possibly both (Tamir, 2009). The emotions that individuals prefer to feel in a given situation may depend on their meta-emotion beliefs, but may also relate to the type of goals that they pursue (Tamir et al., 2007; Tamir, 2011). Different goals have contrasting psychophysiological demands, so it is reasonable to expect that individuals will vary in how they want to feel when pursuing different goals (Tamir, 2009). Additionally, different goals may necessitate certain emotional experiences. Anger may promote confrontational goals, and fear may be useful if the goal is to avoid danger. Some emotions are perhaps more suitable than others depending on which ones can help an individual to better cope with the demands of a particular goal. If individuals pursue a hedonic goal (i.e., a short-term goal which may offer immediate benefit), then they may prefer to experience emotions that maximise immediate, hedonic rewards (Tamir et al., 2007). Conversely, if individuals pursue an instrumental goal (i.e., a long-term goal which promotes delayed rather than immediate benefit) they may forego immediate hedonic rewards, and prefer to experience emotions that promote the attainment of that goal (Mischel et al., 1989; Tamir, 2009). There are many real-life scenarios in which this process can be observed. For example, studying for an exam is generally not considered pleasant. Students persevere in their studies nonetheless, for the sake of long-term future academic success (Tamir, 2009). Although hedonism and motivation are logically connected, there are reasons for behaviour other than immediate reward (Welch and Adams, 2003; Lewis and Sullivan, 2005). Regulating emotions to promote the attainment of long-term, possibly challenging goals may involve prioritizing unpleasant emotions. After all, regulated pleasant emotions may be useful as well as pleasant, whereas regulated unpleasant emotions may only be useful.

### Emotion Regulation in Musical Practice

The current study investigates the principles of hedonic and instrumental emotion regulation in the context of musical practice. Research on this topic is scarce. Literature on musical practice has more commonly focused on strategies specifically for learning or playing music, whereas literature on emotion regulation has often ignored musical practice as an applied domain. Furthermore, music and emotion research often remains concerned with the regulation of emotion *by* music. This literature notes a range of psychobiological benefits associated with listening to music and the use of music as a device for emotion regulation. Benefits include the reduction of stress, improved mood, and protection against mental and physical illness (e.g., Thayer et al., 1994; Juslin and Västfjäll, 2008; Van Goethem and Sloboda, 2011; Saarikalio et al., 2012; Thoma et al., 2013). However, these connections have often been based on samples consisting of non-active musicians. Only a small quantity of research has concentrated on the emotional behaviour of musicians specifically. Some of this research has focused on the emotional consequences of music-making. Findings in this area suggest that, for example, choir singing can positively impact emotional state, immune competence and social connectedness (Kreutz et al., 2005; Bullack et al., 2018). More recently, Peistaraite and Clark (2020) have investigated emotion regulation in the context of self-regulated learning (SRL) processes among classical musicians. This study demonstrated that a musician is more likely to engage in SRL when they had a higher use of emotion reappraisal which is considered to be an adaptive regulation strategy.

Gross (1999) stresses the importance of proper emotion regulation, particularly as emotions can arise during moments of challenge or opportunity. If our musical practice experiences are to be positive and productive, then we argue that the emotion regulation behaviour of musicians in the context of musical practice is seen as an important issue. After all, musical practice is valued by musicians for its many benefits (Austin et al., 2006). Some musicians may be focused on short-term benefits, such as practicing music because it is enjoyable or because it provides some kind of psycho-social-economic reward. Other musicians may be focused on long-term benefits, such as mastering musical and instrumental skills over a longer period of time. Despite its many benefits, musical practice is not always considered pleasant, nor does it necessarily yield immediate progress (Ericsson et al., 1993). Depending on their specific goals, musicians may persevere despite the challenging reality of practice. Presumably then, immediate hedonic emotional reward is not the only driving force behind musicians’ motivation to practice music. We expect that musicians will adopt specific, regulated emotional stances which (i) promote the attainment of their practice goals, and (ii) are consistent with their meta-emotion beliefs regarding the functional impact that different emotions may have on their practice. By means of an online questionnaire, three specific hypotheses were investigated in this study:

**H_1_**: Musicians will report using affect-improvement strategies more often than affect-worsening strategies in order to influence how they feel during their musical practice.

**H_2_**: Greater reported use of affect-worsening strategies will be associated with stronger meta-emotion beliefs supporting the possible benefits of unpleasant emotions in musical practice.

**H_3_**: In contrast to musicians who pursue short-term goals, musicians who pursue long-term goals will (1) hold stronger meta-emotion beliefs supporting the utility of unpleasant emotions in musical practice, and (2) may seek to experience emotions in musical practice which do not solely emphasise immediate hedonic reward.

## Materials and Methods

### Participants

Participants (*N* = 421; female = 254) took part in response to a study advertisement which was emailed to a range of music institutions around the world, including professional orchestras, conservatoires, music universities, and others. The majority of participants were recruited from: United States (113), United Kingdom (72), Germany (64), Norway (42), Austria (26), Denmark (14), Ireland (14), all other participating countries (≤10 each).

### Materials and Procedure

Ethical approval for this study was provided by the responsible institutional review board (see the section “Ethics Statement”). Participants provided informed consent to take part, and then completed an English online questionnaire seeking information on the following topics:

#### Demographics and Musical Background

Participants reported their age and the age at which they began playing music (Age of Commencement; AoC). From these the total number of Years of Playing (YoP) was calculated. Participants were also asked what musical instruments they play, and how actively they are involved in a range of musical styles. In addition, they provided information about their musical practice times: Current weekly musical practice was assessed by asking participants how many hours per week and days per week they practice music. Cumulative Life Practice time (CLP) was derived from year-by-year weekly practice hours, according to a retrospective self-report. In order to gauge participants’ overall attitude toward musical practice, three items were taken from the Flow State Scale (Jackson and Marsh, 1996). Using a seven point Likert scale (1 = strongly disagree… 7 = strongly agree), participants first indicated how much they enjoy the experience of musical practice, second, how focused they are on what they are doing during practice, and third, the extent to which they feel being in control of their musical practice.

#### Intrapersonal Emotion Regulation Strategies

Participants were asked to report how often they used different emotion regulation strategies to influence how they felt over the last 2 weeks of their musical practice. Participants were not asked to report how *effective* each strategy was. Instead, they reported only the extent to which each strategy was used during musical practice. A similar approach was used by Lane et al. (2011). Items were adapted from the EROS scale (Emotion Regulation of Others and Self; Niven et al., 2011) and included two sub-scales: affect-improvement (strategies intended to bring about pleasant emotions; e.g., “I thought of positive aspects of my situation to try to improve how I felt”) and affect-worsening (strategies intended to bring about unpleasant emotions; e.g., “I thought about negative experiences to try to make myself feel worse”). The Cronbach’s α-coefficients was 0.74 for affect-improvement strategies and 0.82 for affect-worsening strategies.

#### Musical Practice Goals

Participants were asked to rate the extent to which they pursue different goals in musical practice (e.g., “It is very important to me to continue to perfect my musical and technical abilities,” “I practice so that I can play a piece exactly as I think it should be”). Items were adapted from Lehmann and Papoušek (2003). See Table 3 for a complete list of practice goal items.

#### Specific Regulated Emotions

Participants completed an emotion scale based on items from the UWIST Mood Adjective Checklist (UMACL; Matthews et al., 1990). First, participants indicated how strongly they typically experienced each of these emotions during musical practice. Second, participants indicated how much they would seek to either increase or decrease the intensity of these same emotions in order to support their musical practice. The specific emotions in this scale were derived from the circumplex model of emotion (Russell, 1980) and assess: pleasant emotions (*Calmness, Happiness*), low-arousal, unpleasant emotions (*Gloom, Downheartedness*), high-arousal, unpleasant emotions (*Anger, Anxiety*) and emotions associated with both high- and low energetic-arousal (*Energy, Nervousness, Sluggishness*). One additional item (*Concentration*) was included in the scale.

#### Meta-Emotion Beliefs

Participants indicated their level of agreement with a custom set of statements concerning the impact of emotions on musical practice (e.g., “Unpleasant emotions such as anger can help get the most out of my practice,” “I must have the right emotional state in order to get the most out of my practice”). See Table 3 for a complete list of meta-emotion belief items.

#### Personality

Participants completed the Ten Item Personality Measure (TIPI; Gosling et al., 2003). This is a brief measure of the Five-Factor Model of personality (Costa and McCrae, 1985). It is measured on a seven point Likert Scale (1 = Disagree Strongly…7 = Agree Strongly). Analysis and findings related to personality will be reported elsewhere.

### Analysis Strategy

The data were analyzed in several steps. In step 1, Principle Component Anaysis (PCA, with oblique rotation) was used to identify potential factors underlying musicians’ meta-emotion beliefs and practice goals, respectively. These factors were then divided into high and low categories, based on a median split. Musicians with a higher belief score were classified as ‘strongly endorsing’ that particular belief, those with a lower belief score were classified as ‘weakly endorsing’ that same belief. Musicians with a higher goal score were classified as having a ‘strong orientation’ toward that particular goal, and those with a lower goal score were classified as having a ‘weak orientation’ for that same goal. In step 2, the high and low categories for meta-emotion beliefs and practice goals were used as between-subjects factors for Multiple Analysis of Variance (MANOVA). MANOVA was used to investigate (A) differences in the emotion regulation strategies used by musicians who hold different meta-emotion beliefs, and (B) differences in the meta-emotion beliefs of musicians who pursue different goals. In step 3, musicians were classified into *ad hoc* subgroups on the basis of their meta-emotion beliefs and goal orientation. Descriptive statistics were used to examine the pattern of specific emotions that these subgroups sought to regulate in order to support their musical practice. Additionally, MANOVA was used to identify differences between these subgroups with respect to the preferred intensity of these emotions. Based on the findings of step 1–3, an additional *post hoc* fourth step of analysis was performed, in which exploratory Categorical Regression (CATREG) was used to investigate demographic and musical experience variables that could potentially account for a musician’s affiliation to one of the aforementioned subgroups. The properties of the data were found to meet the assumptions required for PCA, MANOVA, and CATREG.

## Results

### Overview of Demographics and Musical Experience

Among the total sample (*n* = 421), the majority of musicians identified as female (*n* = 254). The mean age of musicians in this sample was 25 years (SD = 8.8; Min/Max = 18/68 years). Numeric information relating to participants’ demographic and musical experience data is shown in Table 1. Bowed string, keyboard, and woodwind instrumentalists were most strongly represented in the sample. Musicians reported Classical or Post-1950s Contemporary Classical as the musical styles in which they were most actively involved. The Shapiro–Wilk test indicated that a number of variables were not normally distributed. Therefore, the Mann–Whitney *U*-test was used in all comparisons for the sake of consistency. Bonferroni corrections for multiple comparisons were made where appropriate.

**TABLE 1:** Descriptive overview of participants’ demographic and musical experience data.

		Professionals (*n* = 120)	Students (*n* = 301)	
		
		Median (IQR) or *n*	Median (IQR) or *n*	Mann–Whitney*U p*-value
Sex (F/M)		60/60	194/107	
Age (years)		31 (26, 42)	21 (20, 23)	*
YoP (years)		25 (19, 35)	15 (12, 17)	*
CLP (1000 h)		18.5 (12, 29)	6.3 (2.5, 10.6)	*
AoC (years)		7 (5, 9)	6 (5, 9)	n.s.^+^
Days		6 (5, 7)	6 (5, 7)	n.s.
Musical styles *(1 = extremely inactive; 7 = extremely active)*				
	Post-1950s contemporary classical	7 (6, 7)	5 (2, 6)	*
	Classical	7 (6, 7)	7 (5, 7)	n.s.
	Jazz	2 (1, 4)	2 (1, 4)	n.s.
	Rock	1 (1, 2)	2 (1, 4)	n.s.
	Pop	1 (1, 3)	2 (1, 4)	n.s.
Primary musical instrument				
	Bowed string	42 (35%)	63 (20.9%)	
	Keyboard	18 (15%)	63 (20.9%)	
	Woodwind	22 (18.3%)	45 (14.9%)	
	Voice	7 (5.8%)	55 (18.3%)	
	Brass	16 (13.3%)	35 (11.6%)	
	Plucked string	12 (10%)	25 (8.3%)	
	Percussion	3 (2.5%)	15 (5%)	

*Bonferroni corrected for multiple comparisons; significant at the p < 0.01 level. ^+^ n.s., not significant. F, female; M, male; AoC, age of commencement; YoP, years of playing; CLP, cumulative life practice time; days, number of days per week currently practicing music; musical styles, primary musical instrument.

### Professional vs. Student Musicians

Participants were asked to identify themselves as either a Professional (*n* = 120) or Student (*n* = 301) musician. The demographic and musical experience data of the sample plausibily differentiates between professionals and students. In general, professionals were older than students and reported longer lifetime involvement with music (as indicated by YoP and CLP). In order to be cognizant of the potential impact of musical experience on subsequent results, preliminary analysis examined possible differences between professionals and students with respect to the emotions typically experienced during musical practice, attitudes toward musical practice, and emotion regulation strategies.

*Emotions typically experienced in musical practice (Likert Scale; 1 = Not at all…7 = A great deal):* The sample as a whole reported typically experiencing Happiness [Median = 5], Energy [5] and Calmness [5] most strongly in musical practice. Unpleasant emotions were not experienced strongly in general. However, student musicians reported experiencing significantly more Gloom [Student Median/Professional Median = 3/2], Sluggishness [3/2], and Anxiety [3/2] compared to professionals (Wilks λ_1,420_ = 0.93, *p* < 0.05, $\eta_{\text{p}}^{2}$ = 0.06).

*Attitudes toward musical practice (Flow State items; Likert Scale; 1 = Strongly disagree…7 = Strongly agree):* a numeric breakdown of participants’ attitudes toward musical practice is shown in Table 2. In general, this sample of musicians reported strong positive attitudes toward musical practice. Compared to students, professionals reported a significantly greater feeling of being in control, and of having greater focus on what they are doing in musical practice.

**TABLE 2:** Musicians’ overall attitudes toward musical practice (1 = strongly disagree… 7 = strongly agree).

	Complete sample	Professionals	Students	
		
	Median (IQR)	Median (IQR)	Median (IQR)	Mann–Whitney *U p*-value
Attitudes to musical practice (flow state items)				
I usually feel in control of what I am doing in my practice	6 (5, 7)	6 (5, 7)	5 (4, 6)	*
I really enjoy the experience of musical practice	6 (5, 7)	6 (5, 7)	6 (5, 7)	n.s.
I am totally focused on what I am doing during in practice	5 (4, 6)	6 (5, 6)	5 (4, 6)	*

*Bonferroni corrected for multiple comparisons; significant at the p < 0.01 level.

*Emotion regulation strategies (Likert Scale; 1 = Not at all…5 = A great deal):* In the overall sample, both affect-improvement and affect-worsening strategies were not used extensively. However, affect-improvement strategies [Median = 1.83] were used more frequently than affect-worsening strategies [Median = 0.75]. There was no difference between professionals and students for the use of affect-improvement strategies. However, professionals used affect-worsening strategies significantly more than students (Wilks λ_1,420_ = 0.69, *p* < 0.05, $\eta_{\text{p}}^{2}$ = 0.06).

In general, differences between professional and student musicians were infrequent. The effect sizes were small in the case of the differences that were observed. As a result, professionals and students were merged into a single sample for the subsequent analysis. Professional vs. student affiliation was re-examined in the later stages of analysis.

### PCA of Meta-Emotion Beliefs and Practice Goals

#### Meta-Emotion Beliefs

PCA with oblique rotation suggested two factors underlying musicians’ meta-emotion beliefs, explaining 52.98% of variance. The first belief factor was named *Emotion-Driven Practice* and explained 29.90% of variance. This factor refers to a musician’s belief that they must have the right emotional state in order to practice effectively, and that they actively seek to experience whatever emotions (positive and/or negative) help them to support their musical practice. The second factor was named *Non-Hedonic Driven Practice* and explained 23.08% of variance. This factor refers to the belief that a musician does not necessarily have to feel good in order to practice effectively, and that unpleasant emotions may help to improve musical practice. These two beliefs include an overlap regarding the possible benefits of unpleasant emotions in musical practice. This overlap is reflected by a weak, positive, statistically significant correlation between the two belief factors (Spearman’s ρ = 0.164, *p* < 0.01). The upper section of Table 3 shows the final rotated factor solution for meta-emotion belief items.

**TABLE 3:** PCA (with oblique rotation) of meta-emotion beliefs and musical practice goals.

	Component	
	Factor 1:	Factor 2:
	*Emotion-Driven Practice*	*Non-Hedonic Driven Practice*
**Meta-emotion beliefs** *(Likert: 1 = strongly disagree…7 = strongly agree)*		
I pay attention to my emotional state during my musical practice	0.76	
I must have the right emotional state in order to get the most out of my practice	0.74	
I actively seek to experience the emotions that I believe will help me improve my practice	0.71	
I know what emotions will help me get the most out of my practice	0.69	
I usually feel that I have to change my emotions in order to get the most out of my practice	0.68	
I don’t always have to feel good in order to practice effectively		0.80
Unpleasant emotions such as anger can help me get the most out of my practice		0.60
I work equally as hard in my musical practice, regardless of how I feel		0.53
Musical practice is best when I am feeling positive		−0.61
I cannot practice well unless I am feeling positive		−0.58
	
	**Factor 1: *Mastery***	**Factor 2: *Enjoyment***
	
**Musical practice goals** *(Likert: 1 = very untrue of me…7 = very true of me)*		
I practice difficult techniques or pieces until I have mastered them	0.84	
It is very important to me to continue to perfect my musical and technical abilities	0.78	
I practice so that I can play a piece exactly as I think it should be	0.75	
I practice my instrument because it helps me relax and forget everything around me		0.87
I practice my instrument for recreation		0.84
I don’t always have to practice difficult pieces, the main thing is that they sound nice		0.60

#### Practice Goals

PCA with oblique rotation suggested two factors underlying musicians’ musical practice goals, explaining 64.54% of variance. The first factor was named *Mastery* and explained 33.55% of variance. This factor refers to practicing music in order to develop expert musical and instrumental skills. The second factor was named *Enjoyment* and explained 30.99% of variance. This factor refers to practicing music for the purposes of enjoyment and recreation. There was a weak, negative, non-significant correlation between these factors (Spearman’s ρ = −0.05, *p* = 0.26), suggesting that these goal pursuits are relatively discrete. The lower section of Table 3 shows the final rotated factor solution for practice goal items.

Each of the meta-emotion belief and practice goal factors were divided into high and low categories using a median-split. Table 4 shows the quantity of musicians assigned to each category, and provides an overview of their demographic and musical experience characteristics. Table 4 indicates that 65% of the musicians identified as professionals were assigned to the high Mastery category, whereas 38% of musicians identified as students were assigned to this category. The following observations are made on a descriptive level only: strong Mastery orientation was characterized by musicians with greater musical experience, as indicated by YoP and CLP. A roughly opposite profile was indicated for strong Enjoyment orientation. This category was populated by 28% of professionals and 48% of students. Furthermore, strong Enjoyment orientation included musicians that were younger, with less musical experience. With respect to meta-emotion beliefs, 59% of professionals and 41% of students showed strong endorsement of Emotion-Driven Practice. However, 75% of professionals strongly endorsed Non-Hedonic Driven Practice, compared to only 33% of students. In addition to this, the strong-endorsement category for both emotion beliefs was populated by older musicians, with greater CLP and YoP.

**TABLE 4:** Overview of the quantity and characteristics of musicians classified into high/low categories for goals and emotion beliefs.

	Practice goals		Meta-emotion beliefs	
	Mastery	Enjoyment	Emotion-Driven Practice	Non-Hedonic Driven Practice
**High**				
No. of musicians assigned	192	176	194	189
Age (median; years)	24	22	23	24
AoC (median; years)	6	7	7	7
YoP (median; years)	17	15	17	18
CLP (median; 1000 h)	10.9	6.7	10.6	11.2
Type (*N*; student/professional)	113/79	143/33	123/71	99/90
**Low**				
No. of musicians assigned	229	245	227	232
Age (median; years)	20	24	22	20
AoC (median; years)	7	6	6	6
YoP (median; years)	16	17	16	13
CLP (median; 1000 h)	6.7	9.8	7.6	7.2
Type (*N*; student/professional)	188/41	158/87	178/49	202/30

*Total sample N = 421; total students n = 301; total professionals n = 120. AoC, age of commencement; YoP, years of playing; CLP, cumulative life practice time.*

### Emotion Regulation Strategies Used by Musicians Holding Different Meta-Emotion Beliefs

#### Emotion-Driven Practice

MANOVA was used to examine the effect of Emotion-Driven Practice beliefs on emotion regulation strategies. Endorsement for this belief (two levels: strong and weak endorsement) was used as a between-subjects factor. There was a significant main effect for belief endorsement (Wilks λ = 6.67; *p* < 0.05; $\eta_{\text{p}}^{2}$ = 0.03) with univariate effects evident for affect-improvement strategies (*F*_1,420_ = 4.49; *p* = 0.03; $\eta_{\text{p}}^{2}$ = 0.01) and affect-worsening strategies (*F*_1,420_ = 9.24; *p* = 0.003; $\eta_{\text{p}}^{2}$ = 0.02). Musicians with strong endorsement of Emotion-Driven Practice reported greater use of affect-worsening strategies and less use of affect-improvement strategies compared to those with weak endorsement of Emotion-Driven Practice beliefs.

#### Non-Hedonic Driven Practice

MANOVA was used to examine the effect of Non-Hedonic Driven Practice beliefs on emotion regulation strategies. Endorsement of this belief (two levels: strong and weak endorsement) was used as a between-subjects factor. There was a significant main effect of belief endorsement (Wilks λ_1,420_ = 16.04; *p* < 0.01; $\eta_{\text{p}}^{2}$ = 0.07) with a univariate effect for affect-worsening strategies (*F*_1,420_ = 30.33; *p* < 0.01; $\eta_{\text{p}}^{2}$ = 0.06), but *not* for affect-improvement strategies. Musicians who strongly endorsed Non-Hedonic Driven Practice reported greater use of affect-worsening strategies.

There was no significant interaction between the two meta-emotion beliefs for either affect-improvement or affect worsening strategies (Wilks λ_1,420_ = 0.34; *p* = 0.71; $\eta_{\text{p}}^{2}$ = 0.02).

### Meta-Emotion Beliefs of Musicians Pursuing Different Goals

#### Mastery Orientation

MANOVA was used to examine the effect of Mastery orientation on meta-emotion beliefs. Mastery orientation (two levels: strong and weak orientation) was used as a between-subjects factor. There was a significant main effect of Mastery orientation (Wilks λ_1,420_ = 45.91; *p* < 0.01; $\eta_{\text{p}}^{2}$ = 0.18) with univariate effects for Emotion-Driven Practice (*F*_1,420_ = 39.86; *p* < 0.01; $\eta_{\text{p}}^{2}$ = 0.08) and Non-Hedonic Driven Practice (*F*_1,420_ = 53.65; *p* < 0.01; $\eta_{\text{p}}^{2}$ = 0.11). Musicians who strongly pursued Mastery goals showed stronger endorsement of both emotion beliefs compared to musicians’ who did not strongly pursue Mastery goals.

#### Enjoyment Orientation

MANOVA was used to examine the effect of Enjoyment orientation on meta-emotion beliefs. Enjoyment orientation (two levels; strong and weak orientation) was used as a between-subjects factor. There was a significant main effect of Enjoyment orientation (Wilks λ_1,420_ = 5.57; *p* < 0.01; $\eta_{\text{p}}^{2}$ = 0.02) with univariate effects for Emotion-Driven Practice (*F*_1,420_ = 3.68; *p* < 0.01; $\eta_{\text{p}}^{2}$ = 0.01) and Non-Hedonic Driven Practice (*F*_1,420_ = 7.65; *p* < 0.01; $\eta_{\text{p}}^{2}$ = 0.01). Musicians who strongly pursued Enjoyment goals showed weaker endorsement of both beliefs, compared to musicians who did not strongly pursue Enjoyment goals.

There was a significant interaction between Mastery and Enjoyment orientation (Wilks λ_1,420_ = 5.99; *p* < 0.01; $\eta_{\text{p}}^{2}$ = 0.02), with a univariate effect evident only for Non-Hedonic Driven Practice (*F*_1,420_ = 12.00; *p* = 0.001; $\eta_{\text{p}}^{2}$ = 0.02), but not for Emotion-Driven Practice. Musicians who strongly pursued Mastery but *not* Enjoyment goals showed the strongest endorsement of Non-Hedonic Driven Practice beliefs compared to musicians who either (A) strongly pursued both, (B) strongly pursued neither, or (C) strongly pursued Enjoyment but *not* Mastery. Musicians with a strong Enjoyment orientation but a weak Mastery orientation showed the weakest endorsement of Non-Hedonic Driven Practice.

### Specific Emotions Regulated in Order to Support Musical Practice

The preceding analyses provided preliminary evidence that the endorsement of Non-Hedonic Driven Practice plays a small, yet conspicuous role in the emotion regulation behaviour of musicians in musical practice. The findings of particular relevance in this regard are summarized:

(a) The weak, positive correlation between Emotion-Driven Practice and Non-Hedonic Driven Practice suggests that some musicians strongly endorsed both. Descriptive statistics confirmed that 100 musicians in this sample strongly endorsed both beliefs.
(b) Strong endorsement of Non-Hedonic Driven Practice was associated with greater reported use of affect-worsening strategies.
(c) Musicians who exclusively pursued Mastery goals (i.e., strong Mastery orientation in addition to weak Enjoyment orientation) showed stronger endorsement of Non-Hedonic Driven Practice compared to musicians with exclusive Enjoyment orientation or musicians with a strong orientation for both Mastery *and* Enjoyment.

Taken together, these findings may indicate that a subgroup of musicians exist whose regulation behaviour could be seen as consistent with instrumental emotion regulation principles. In order to strengthen this perspective, we addressed the question of whether certain musicians selected to experience unpleasant emotions in order to support their musical practice. Two contrasting subgroups were derived on the basis of musicians’ goals and emotion beliefs. Musicians were assigned into one of two subgroups upon fulfilling the criteria in Table 5. A brief summary of the demographic and musical experience characteristics of these subgroups is shown in Table 6.

**TABLE 5:** Criteria for subgroup classification.

	Subgroup 1	Subgroup 2
**Practice goal**	Strong Mastery orientation Weak Enjoyment orientation	Weak Mastery orientation Strong Enjoyment orientation
**Meta-emotion belief**	Strongly endorsing Non-Hedonic Driven Practice	Weakly endorsing Non-Hedonic Driven Practice
**Subgroup code**	“Mastery*Non-Hedonic”; M*NH	“Enjoyment*Positive”; E*P

**TABLE 6:** Demographic and musical expertise characteristics of subgroup musicians.

	Mastery*Non-Hedonic M*NH (*n* = 84)	Enjoyment*Positive E*P (*n* = 72)
Status (*N*; student / professional)	27/57	61/11
Age (median; years)*	28	22
AoC (median; years)	7	7
YoP (median; years)*	21	15
CLP (median; 1000 h)*	15.8	5.6
Days*	6	5

*Bonferroni corrected for multiple comparisons; significant at the p < 0.01 level. AoC, age of commencement; YoP, years of playing; CLP, cumulative life practice time; Days, number of days per week currently practicing music.

**FIGURE 1:**
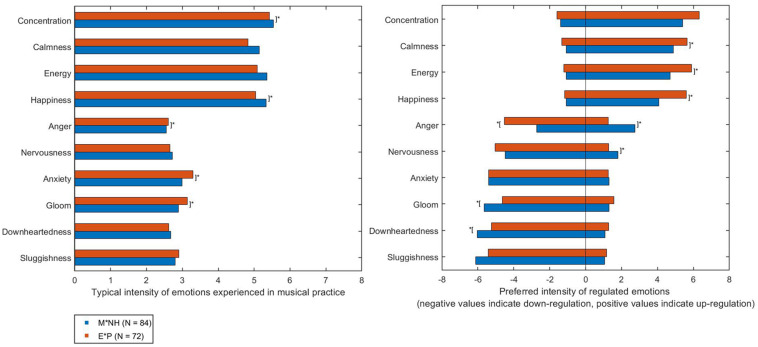
Mean ratings for typical (left side plot) and regulated (right side plot) emotions in musical practice. Emotions were rated on a seven-point Likert scale (1 = not at all…7 = a great deal). Square brackets marked * indicate statistically significant differences between subgroups emotion ratings (Significant at the *p* < 0.01 level).

Descriptive statistics were examined to investigate the intensity of emotions that were typically experienced by both subgroups. An overview of these emotions can be seen in Table 7 and Figure 1 (left side plot). Both subgroups showed a relatively similar overall profile of these emotions; Concentration, Calmness, Energy and Happiness were experienced most strongly. Unpleasant emotions such as Downheartedness and Sluggishness were not experienced strongly in general. However, the M^∗^NH subgroup reported experiencing significantly more Concentration and Happiness in their musical practice, whereas the E^∗^P typically experienced greater levels of Anger, Anxiety, and Gloom. The quantity of students assigned to the E^∗^P group may account for the greater levels of these unpleasant emotions. Students reported less positive attitudes to musical practice; see Table 2). The extent to which musicians in these subgroups sought to regulate these emotions is shown in Figure 1 (right side plot).

**TABLE 7:** Intensity of emotions typically experienced by subgroups in musical practice (Likert Scale; 1 = Not at all… 7 = A great deal).

	Mastery*Non-Hedonic M*NH (*n* = 84)	Enjoyment* Positive E*P (*n* = 72)	Mann–Whitney *U p*-value	Direction
			
	Median (IQR)	Median (IQR)		
Concentration	6 (5, 7)	5 (5, 6)	*	M*NH > E*P
Calmness	5 (4, 6)	5 (5, 6)	n.s.	–
Energy	5 (3, 6)	5 (5, 6)	n.s.	–
Happiness	4 (4, 5)	5 (5, 6)	*	E*P > M*NH
Anger	3 (2, 4)	2 (1, 3)	*	M*NH > E*P
Nervousness	2 (1, 3)	2 (1, 3)	n.s.	–
Anxiety	2 (1, 3)	3 (2, 4)	*	E*P > M*NH
Gloom	2 (1, 2)	3 (2, 5)	*	E*P > M*NH
Downheartedness	2 (2, 3)	2 (1, 4)	n.s.	–
Sluggishness	2 (1, 3)	3 (2, 4)	n.s.	–

*Bonferroni corrected for multiple comparisons; significant at the p < 0.01 level.

Both subgroups reported that they would seek to substantially increase the intensity of Concentration, Energy, Calmness, and Happiness to support their musical practice. The E^∗^P subgroup preferred to increase these emotions to a greater extent than the M^∗^NH subgroup. Both subgroups also reported that they would seek to substantially reduce the intensity of Anxiety, Gloom, Downheartedness and Sluggishness, despite only moderately intense typical experiences of these emotions. The M^∗^NH subgroup generally sought to decrease these unpleasant emotions to a greater extent than the E^∗^P subgroup. Differences in subgroup regulation were additionally seen with respect to Anger and Nervousness. The M^∗^NH subgroup reported they would seek to increase the intensity of Anger and Nervousness to a significantly greater extent than the E^∗^P subgroup. M^∗^NH also preferred to reduce Anger significantly less than the E^∗^P subgroup. MANOVA using musicians in the two subgroups only indicated a significant main effect of subgroup affiliation (M^∗^NH or E^∗^P; Wilks λ_1,420_ = 14.42; *p* < 0.01; $\eta_{\text{p}}^{2}$ = 0.50) with univariate effects observed for both the up- and down-regulation of several emotions (see Table 8 for a summary).

**TABLE 8:** Overview of specific emotion regulations (up-regulation or down-regulation) reported by M*NH and E*P subgroups.

Emotions up-regulated	$\eta_{\text{p}}^{2}$	*p*	Direction
Energy	0.11	*	E*P > M*NH
Calmness	0.05	*	E*P > M*NH
Happiness	0.18	*	E*P > M*NH
Anger	0.39	*	M*NH > E*P
Nervousness	0.06	*	M*NH > E*P
**Emotions down-regulated**			
Anger	0.14	*	E*P > M*NH
Gloom	0.04	*	M*NH > E*P
Downheartedness	0.03	*	M*NH > E*P

*Bonferroni corrected for multiple comparisons; significant at the p < 0.01 level.

### The Musical Background of Subgroups

An exploratory Categorical Regression (CATREG; Meulmann and Heiser, 1999) was used to investigate demographic and musical experience characteristics that could account for a musician’s affiliation to one of the subgroups. CATREG is advantageous in this context as it can be performed with little reliance on the assumptions required for standard multiple regression, including normality, multicollinearity, and homogeneity of variance (Shrestra, 2009). It can also be performed when the predictor and outcome variable(s) are any combination of continuous, ordinal or categorical. As part of the CATREG process, all variables are assigned an optimal scaling. The optimal scale is most often consistent with a variable’s data type. In the current analysis, subgroup affiliation was scaled as a categorical target variable. Predictor variables included demographics, musical experience variables, attitudes toward musical practice (Flow), and ratings of typical emotion experiences. All predictor variables were scaled as either continuous or ordinal (on the basis of the practical implication between the levels of each variable). The CATREG was performed in two steps. In step 1, the Lasso (Least Absolute Shrinkage and Selection Operator; Tibshirani, 1996) regularization method was used to identify predictors with the greatest capacity to predict subgroup affiliation. In step 2, a simplified version of the regression was re-run, using only the variables identified by the Lasso as the optimal subset of predictors. A similar format for reporting CATREG has been utilized in previous studies (Schmidt et al., 2019).

The final regression model was statistically significant (*F*_9,155_ = 9.12; *p* < 0.01) explaining 58.6% of variance. A set of eight variables was identified by the Lasso as the most parsimonious set of predictors. These variables were brought forward into the final, simplified model. Affiliation to the M^∗^NH subgroup (with E^∗^P affiliation as the reference category) was predicted by: Professional musician status, older age, more days per week playing an instrument, greater reports of feeling completely focused in musical practice, and stronger typical experiences of Concentration and Anger. Accordingly, affiliation to the E^∗^P subgroup was associated with Student musician status, younger age, fewer days playing an instrument, less feelings of focus in practice, and stronger typical experience of Happiness and Gloom. The contribution of each variable in the final model is shown in Table 9.

**TABLE 9:** Variables included in the final CATREG model.

Target = subgroup affiliation (E*P = 0; M*NH = 1)	B -coefficient	*p*
Status *(student = 0; professional = 1)*	0.21	*
Age	0.20	*
Days *(number of days per week currently practicing music)*	0.23	*
Feeling completely focused in practice (*flow state)*	0.34	*
Typical intensity of happiness	–0.19	*
Typical intensity of gloom	–0.29	*
Typical intensity of anger	0.18	*
Typical intensity of concentration	0.16	*

*Significant at the p < 0.05 level. Adjusted R^2^ = 0.58.

## Discussion

Via an online questionnaire distributed to musicians, this study investigated emotion regulation behaviours in the context of musical practice. With respect to the hypotheses outlined in the introduction, the following summary is provided:

**Findings support H_1_:** Musicians reported using affect-improvement strategies more often than affect-worsening strategies to influence how they felt during their musical practice.

**Findings support H_2_:** Greater reported use of affect-worsening strategies was associated with stronger endorsement of the meta-emotion belief *Non-Hedonic Driven Practice*. A component of this belief relates to the possible benefits of unpleasant emotions.

**Findings support H_3_:** Musicians who strongly pursued long-term Mastery goals showed stronger endorsement of Non-Hedonic Driven Practice in contrast to musicians who strongly pursued short-term Enjoyment goals. Additionally, some musicians in the M^∗^NH subgroup (i.e., musicians with strong Mastery orientation *and* strong endorsement of Non-Hedonic Driven Practice) sought to increase the intensity of Anger and Nervousness in order to support their musical practice. These emotions were targeted in conjunction with several pleasant emotions.

**FIGURE 2:**
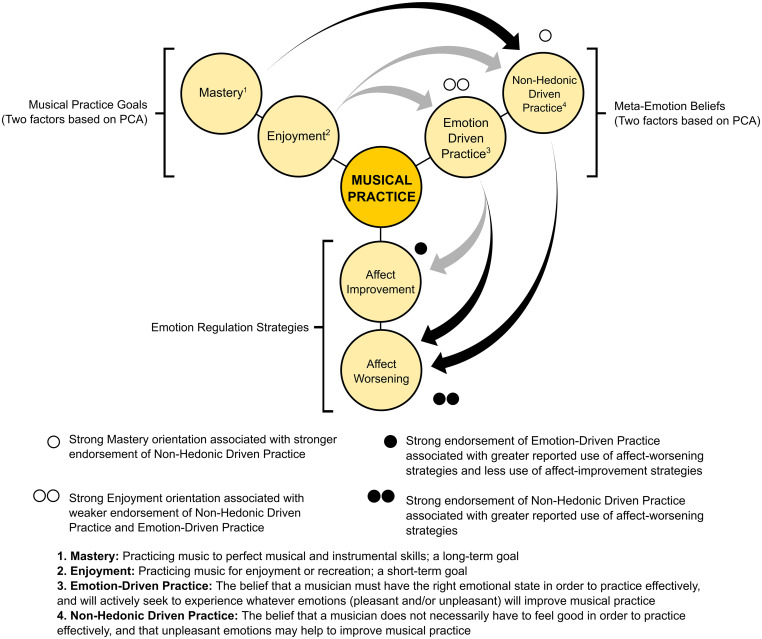
Overview of the main concepts under investigation (practice goals, meta-emotion beliefs, and emotion regulation strategies), and a selection of relevant results. Arrows originate from the independent variables (between-groups factors) to the dependent variables used in MANOVAs (see sections “Emotion Regulation Strategies Used by Musicians Holding Different Meta-Emotion Beliefs’ and “Meta-Emotion Beliefs of Musicians Pursuing Different Goals”). The colour of the arrows indicate the direction of univariate effects (e.g., Black = strong goal orientation associated with stronger belief endorsement; Grey = strong goal orientation associated with weaker belief endorsement).

The findings of this study suggest complex potential connections between musical practice goals, meta-emotion beliefs, and emotion regulation strategies. A selection of these connections are shown in Figure 2. Findings of this kind have not been demonstrated previously in the context of musical practice. Nonetheless, there are a number of important connections to existing research on music-making, as well as a range of other topics. Most notably, our findings complement research conducted by Peistaraite and Clark (2020). They investigated how emotion regulation processes relate to self-regulated learning (SRL) among classical musicians. In this context, emotion regulation behaviours were framed as general cognitive-affective behaviours. Their findings showed that reappraisal (an emotion regulation process that involves modifying the way in which a situation is evaluated; Gross and John, 2003) correlated with multiple scales of all three phases of the SRL construct: forethought, performance, and self-reflection (according to Zimmerman, 2002). Peistaraite and Clark (2020) showed that reappraisal was associated with enhanced use of SRL in musicians and suggested the potential utility in considering emotion regulation as part of SRL in musicians. Our approach complements their findings by assessing self-reported regulation of specific targeted emotions during the musical practice process.

*Musical practice goals:* PCA identified two factors underlying musicians’ practice goals: Mastery and Enjoyment. Mastery, i.e., practicing music in order to master musical and instrumental skills is a long-term goal that is pursued by musicians who are serious about their craft. Enjoyment, i.e., practicing music for enjoyment or recreation is a short-term goal which can offer immediate hedonic emotional rewards. Similar goals pursuits have been discussed and contrasted with one another in a variety of contexts including self-selected performance goals (Lehmann and Papoušek, 2003), deliberate practice (Ericsson et al., 1993), musical ability and identity (McPherson et al., 2012), and also in sport and exercise (Karageorghis, 2017). The current Mastery and Enjoyment goal factors were weakly correlated, suggesting that these goals are relatively discrete.

*Emotion regulation strategies and meta-emotion beliefs:* There is a coherent relationship between the strategies musicians reported using to regulate their emotional state, and their beliefs regarding the functional impact of emotions in musical practice. If individuals believe that certain emotions are helpful to the pursuit of a particular goal, then it is reasonable to expect that whatever emotion regulation strategy they use is intended to bring about these desired emotions (Lane et al., 2011). Current findings showed that stronger endorsement of Emotion-Driven Practice was associated with greater reported use of affect-worsening strategies and significantly less reported use of affect-improvement strategies. Given that affect-improvement strategies are intended to bring about pleasant emotions, the intensity to which pleasant emotions were typically experienced in musical practice may help to interpret these findings. Generally, musicians experienced pleasant emotions quite strongly in their musical practice, but not unpleasant emotions. Presumably, musicians will not need to regulate every desired emotion. Some emotions may already be at the desired or optimal intensity (Lane, 2008). Musicians who strongly endorsed Emotion-Driven Practice may not have used affect-improvement strategies more often because pleasant emotions might have already been experienced at a sufficient intensity. The use of affect-worsening strategies may function as a counterbalance, by ensuring that these emotions remain at the preferred intensity.

Stronger endorsement of Non-Hedonic Driven Practice was associated with greater reported use of affect-worsening strategies. Endorsement of this belief had no effect on the reported use of affect-improvement strategies. A component of Non-Hedonic Driven Practice specifically concerns the potential benefit of unpleasant emotions in musical practice. This finding suggests that musicians who believe that unpleasant emotions may help to improve musical practice may be willing to experience an unpleasant emotional state. This result is consistent with findings from Lane et al. (2011) who demonstrated that athletes who held the belief that increasing anger and/or anxiety would improve running performance reported using strategies to increase these same emotions.

*Meta-emotion beliefs and practice goal orientation:* Strong Enjoyment orientation was associated with weaker endorsement of Emotion-Driven Practice and Non-Hedonic Driven Practice. In contrast, strong Mastery orientation was associated with stronger endorsement of Non-Hedonic Driven Practice. There was no effect of Mastery orientation on endorsement for Emotion-Driven Practice. Research shows that Mastery-oriented musicians use a more diverse set of learning strategies in their musical practice (Lehmann and Papoušek, 2003). Associations between performance and the functional influence of emotions develop over the course of time and experience (Hanin, 2010). Therefore, in addition to learning strategies, it is plausible that Mastery-oriented musicians may also possess a more diverse set of meta-emotion beliefs. Endorsement of Non-Hedonic Driven Practice may arise as a consequence of greater exposure to the challenges of mastering musical and instrumental skills. Indeed, the Mastery-oriented musicians in the current sample were more experienced and reported a greater Cumulative Life Practice time (CLP) compared to Enjoyment-oriented musicians (strong endorsement, respectively) on a descriptive level (see Table 4). It is possible that Mastery-oriented musicians may be driven, to at least some degree, by instrumental motives for regulating emotions. These musicians may be more willing to experience unpleasant emotions if they are believed to be beneficial to the development of expert musical skills.

*Regulating specific emotions to support musical practice:* The theoretical framework outlined in the introduction provides information concerning the specific performance benefits associated with different emotional states. This information is discussed here with respect to the specific emotions that musicians sought to regulate in their musical practice. Musicians were assigned into subgroups on the combined basis of their practice goals and meta-emotion beliefs. This allowed contrasting approaches to musical practice to be compared. The first subgroup (M^∗^NH) comprised musicians with a strong Mastery orientation, weak Enjoyment orientation, and who strongly endorsed Non-Hedonic Driven Practice. The second subgroup (E^∗^P) comprised musicians with a strong Enjoyment orientation, weak Mastery orientation, and who did not strongly endorse Non-Hedonic Driven Practice. When asked to report how they would regulate specific emotions in order to support their practice, points of convergence and contrast between these subgroups were observed.

*(1) Avoidance of unpleasant, low-arousal emotions:* Both subgroups sought to down-regulate the intensity of Gloom, Sluggishness, and Downheartedness. Although these emotions were typically experienced at a relatively low intensity, the preferred intensity was very low. Down-regulating these emotions may be advantageous for a musician as they are associated with an inability to successfully regulate other emotions - emotions which may be more useful (Kaleńska–Rodzaj, 2018). Furthermore, the passive quality of these emotions may prompt behavioural responses that are not conducive to effective musical practice (Gross, 1999). Practice may be better supported by emotions that prompt a musician to be proactive.

*(2) Prioritizing pleasant emotions:* Both subgroups sought to up-regulate the intensity of Happiness, Energy, and Calmness. Not only were these emotions typically experienced quite strongly in musical practice, but the preferred intensity was also very high. Pleasant emotions are often linked to improved performance (for a summary in sport contexts, see Lane, 2012). A substantial up-regulation of these emotions may be advantageous for a musician, as even mild increases in positive affect has been associated with better performance on creative, academic, and problem-solving tasks (e.g., Carnevale and Isen, 1986; Isen et al., 1987; Estrada et al., 1994; Ashby et al., 1999; Carmona-Halty et al., 2018). Furthermore, practicing music with joy has been associated with faster and more enduring adaptation processes when dopamine is released in the limbic system of the brain (Altenmüller and Jabusch, 2014).

*(3) Increasing low-valence, high-arousal emotions:* Unlike the E^∗^P subgroup, some M^∗^NH musicians sought to up-regulate Anger and Nervousness to a moderate intensity. Increasing Anger and Nervousness was not widespread in the M^∗^NH subgroup however – some M^∗^NH musicians also sought to decrease the intensity of these same emotions. In that sense, this finding is comparable to findings of Lane et al. (2011) who reported that a minority of runners believed that increasing unpleasant emotions would improve running performance. Unpleasant emotions are generally not associated with improved performance and practice. Unpleasant emotions may have a debilitating influence on concentration (Hanton et al., 2000; Martinent and Ferrand, 2009), and they may also be characterized by undesirable physical features such as trembling, anxiety and sickness (Kaleńska–Rodzaj, 2018). However, as previously mentioned, unpleasant emotions may sometimes be more helpful than pleasant emotions (Davis et al., 2010), although this may depend on the extent of these emotions (Yerkes and Dodson, 1908). Furthermore, anger does not necessarily lead to aggression, nor nervousness to anxiety or stress (Deffenbacher and McKay, 2000). Unpleasant emotions can also be tempered by pleasant emotions (Cantón and Checa, 2012). As above, the M^∗^NH subgroup also sought to up-regulate Happiness, Energy and Calmness. If Anger and Nervousness are up-regulated alongside these pleasant emotions, this may in fact represent an advantage to having a strong Mastery orientation in musical practice.

*The mixed mindset of Mastery:* Unpleasant emotions are not simply the opposite of pleasant ones. They differ from one another in terms of their behavioural and cognitive effects, and there are independent neural substrates that allow both to be experienced simultaneously (George et al., 1995; Norman et al., 2011). If pleasant and unpleasant emotions are experienced together, this is referred to as a mixed emotional state (Carrera and Oceja, 2007). Bowed-string student instrumentalists reported experiencing mixed emotional states whilst waiting to perform onstage. In this context, feelings of hope and joy were combined with feelings of sadness and anxiety (Kaleńska–Rodzaj, 2018). Mixed emotional states may involve experiencing one emotion more intensely than another (Carrera and Oceja, 2007). This corresponds to the manner in which emotions were up-regulated by the M^∗^NH subgroup. These musicians sought to up-regulate Happiness, Energy, Calmness, Anger, and Nervousness. The three pleasant emotions in this set were up-regulated to a greater intensity than the two unpleasant emotions, on a descriptive level. Considering that the M^∗^NH musicians had greater musical experience than the E^∗^P musicians, strongly up-regulating pleasant emotions in conjunction with moderate up-regulation of unpleasant emotions may be a regulatory decision that longer lifetime involvement with music has brought to light. It is also possible that seeking a mixed-emotional state may support the long-term development of musical and instrumental skills, considering that the majority of M^∗^NH musicians also identified as professionals (see Table 6). A mixed-emotional state may result in ‘approach-avoidance’ conflict (Dollard and Miller, 1969). This conflict is said to increase an individual’s focus, and give a task greater personal significance. In the case of Mastery-oriented musicians, a mixed-emotional state may actually be preferable to an exclusively pleasant or exclusively unpleasant emotional state (Mukherjee et al., 2012).

*Implications for musicians’ health:* Affect-improvement strategies and pleasant emotions were prioritized *in general* in musical practice. However, some musicians targeted unpleasant emotions in order to support the pursuit of their Mastery goals. With this in mind, there is a health and well-being perspective to be considered, especially in light of the challenging reality of musicianship. Developing musical expertise is no easy task. It requires many years of intense, deliberate practice (Ericsson et al., 1993). Even then, expertise is not guaranteed. There are many challenges that can negatively impact the health of a musician, causing them to suffer, or quit altogether. Musicians then, in addition to developing expert musical skills, may wish to pursue expertise regarding their own health and how they may influence it (Spahn et al., 2004). Unpleasant emotions can negatively impact psychological health (Tamir et al., 2008) and is associated with a range of psychopathologies including depression and stress (Du et al., 2018). Music students have been shown to experience significantly greater levels of depression and anxiety compared to other student groups (Spahn et al., 2004). Additionally, musicians with focal dystonia (commonly referred to as musician’s cramp; a loss of voluntary motor control during task-specific movements such as playing a musical instrument) are six times more likely to have elevated anxiety, perfectionism, and stress characteristics compared to healthy musicians (Ioannou and Altenmüller, 2014). Several researchers have suggested that these characteristics could be aggravating risk factors in the development of dystonia (Jabusch and Altenmüller, 2004; Jabusch et al., 2004; Enders et al., 2011). Furthermore, unpleasant emotions are also known to modulate motor function (Ioannou et al., 2016), and may be accompanied by muscular tension. Excessive muscular tension is a known risk-factor for musicians. Up to 73.5% of musicians in an orchestral sample reported that excessive muscular tension was an important contributor to developing pain or injury (Ackermann et al., 2012). The situation is therefore complex for musicians: On one hand, some musicians may seek out unpleasant emotions to support the pursuit of their goals. On the other hand, unpleasant emotional experiences may put a musician at greater risk of psychological or physical harm.

### Strengths and Limitations

There are several strengths to this study. First, the emotion scale allowed participants to express their desired intensity of different emotions. Previous research has often focused only on whether an individual seeks to increase or decrease their emotions relative to their current emotional state, without identifying the *optimal* or *preferred* state (Lane et al., 2011). A second strength comes from the use of the EROS scale to examine intrapersonal emotion regulation behaviours. The EROS scale was not specifically developed for use in musical contexts. The use of a domain-general scale enables comparisons to be made between the emotion regulation strategies used by different populations and in different contexts. This topic is of long-standing interest to emotion researchers (Baumeister and Heatherton, 1997; Gross, 1999).

This study has a number of limitations. First, although a structured questionnaire was used, certain modules have not been validated, and thus may produce contrasting results in different samples. Second, the invitation to participate in this study was distributed to musicians indirectly via the musicians’ host institute. As such, this study was advertised to an unknown total number of musicians. The disadvantage of this approach is that the response rate cannot be determined. Given that all participants were volunteers and not chosen, we acknowledge that the individuals that decided to take part may have had a specific attitude resulting in an impact on the findings. Third, emotion regulation behaviours were self-reported and retrospective. This type of data may be inaccurate due to an individual’s level of self-knowledge or a desire to portray themselves more favourably. However, Jabusch et al. (2009) note that retrospective self-reporting may be a reliable method for assessing musical activities such as accumulated practice time. For example, Lehmann and Ericsson (1998) found a strong correlation between a musician’s practice diary and retrospective reports of practice, with an estimated error of only 10–15%. In the absence of precise information concerning the accuracy of retrospectively reported emotion regulation behaviour, we acknowledge the possibility of an equivalent level of error in these data. Fourth, there was a moderate over-representation of females in the sample (56%). It may be advisable to maintain a gender-balanced sample in order to avoid potential confounds related to gender. That being said, the over-representation of females in this sample is not considered to have impacted the current findings. Females are similarly over-represented in a variety of German music degree programs (German Music Information Centre, 2021), and also in a range of music ensembles in the United States (Elpus, 2014). Fifth, this study did not include any measure of practice outcomes. As a consequence, this study cannot claim specific performance advantages associated with any particular emotion regulation behaviours.

### Directions for Development

It may be beneficial for musicians and music educators to understand whether there are specific regulated emotional states which can support specific musical outcomes, either immediate and/or long-term. Strategies that may bring about these emotional states could then be integrated into musicians’ practice activities on the basis of their preferred outcomes. Future research should also focus on the development of musicians’ meta-emotion beliefs. Musical practice involves repetitive activities, so it is plausible that a musician’s experiences during practice develop into stable patterns of thoughts and behaviour. Reflecting on these patterns with respect to their outcomes may lead to the development of meta-emotion beliefs. Understanding the development of these beliefs may help educators to provide more appropriate guidance to students. Future research may wish to examine the relationship between emotion regulation strategies and goal orientation. The effective use of emotion regulation strategies may reinforce musicians’ goals, whereas the ineffective use of regulation strategies may result in musicians adjusting their goals unnecessarily (Mazur and Laguna, 2019). A similar sentiment is made by Peistaraite and Clark (2020), who note that musicians may avoid setting learning goals or may set very vague goals if they experience strong negative emotions which they cannot handle every time their goal is challenged. Finally, research could focus on the relationship between musicians’ health and emotion regulation behaviours, with a view to maximising health as a long-term outcome.

## Conclusion

This study investigated emotion regulation behaviour in the context of musical practice. Findings suggest that musicians engage in emotion regulation behaviours to support the pursuit of their preferred musical practice goals. Generally, musicians reported using affect-improvement strategies more often than affect-worsening strategies in order to influence how they felt during musical practice. However, musicians with stronger Mastery orientation may believe that unpleasant emotions can improve musical practice. In terms of specific targeted emotions, these musicians may be willing to up-regulate unpleasant emotions to support their pursuit of musical and instrumental skills. This is seemingly not the case for musicians with a strong Enjoyment orientation, who preferred to up-regulate only pleasant emotions to support their practice. The extent of lifetime involvement in music may influence these regulating behaviours. It is hoped that this study and future research on this topic will provide musicians with novel skills for better pursuit of their practice goals and will help to maximize health and well-being in musical practice.

## Data Availability Statement

The datasets for this article are not publicly available because public availability of data is not covered by the ethics application and the ethics approval provided for this study (Institutional Review Board of the State Chamber of Physicians of Saxony IRB 425931; Application reference: EK-BR-73/19-1). Requests to access the datasets should be directed to GBM (gerard.madden@mailbox.hfmdd.de).

## Ethics Statement

The studies involving human participants were reviewed and approved by the Institutional Review Board of the State Chamber of Physicians of Saxony (IRB 425931; Application reference: EK-BR-73/19-1). The Participants provided their written informed consent to participate in this study.

## Author Contributions

GBM conceived, designed and conducted the study, performed the statistical analysis, and wrote and reviewed the manuscript. H-CJ designed the study, discussed the statistical analysis, and wrote and reviewed the manuscript. All authors contributed to the article and approved the submitted version.

## Conflict of Interest

The authors declare that the research was conducted in the absence of any commercial or financial relationships that could be construed as a potential conflict of interest.
